# Prescribing and Monitoring of High Dose Antipsychotic Therapy (HDAT) in the Acute Inpatient Setting

**DOI:** 10.1192/bjo.2022.452

**Published:** 2022-06-20

**Authors:** Gemma Buston, Helen Kay, Ife Nwibe, Oliver Edge, Oli Sparasci, Amit Sindhi

**Affiliations:** 1 Greater Manchester Mental Health NHS Foundation Trust, Greater Manchester, United Kingdom; 2 Pennine Care NHS Foundation Trust, Greater Manchester, United Kingdom

## Abstract

**Aims:**

High Dose Antipsychotic Therapy (HDAT) is defined by the Royal College of Psychiatrists as either: a total daily dose of a single antipsychotic which exceeds the upper limit stated in the BNF, or a total daily dose of two or more antipsychotics which exceeds the BNF maximum calculated by percentage. HDAT is defined as ‘off-label’ prescribing and the prescribing clinician should clearly document rationale for its prescription and clear discussion with the patient regarding the risks and benefits. If the patient is deemed to lack capacity, this should be clearly documented, and appropriate legal processes followed as defined by the Mental Health Act 1983. The use of HDAT comes with greater risk of physical health complications and requires regular monitoring of electrocardiogram (ECG), body mass index (BMI) and blood biochemistry. Aims: To re-audit the number of inpatients prescribed HDAT across three acute general adult inpatient wards, and to establish whether guidelines for the prescribing and monitoring of HDAT are adhered to.

**Methods:**

Initial audit was completed in January 2020. Education sessions were provided to rotational junior doctors in the six months following initial audit. For re-audit, medication cards for each patient on the electronic bed-state at 9pm on 27/11/2021 were checked for HDAT prescription. Data were collected from electronic notes of patients identified as being on HDAT.

**Results:**

Initial audit in 2020 demonstrated that 3 of 49 inpatients (6%) were prescribed HDAT, with no evidence of documentation of rationale, and variable monitoring of physical health indicators. Re-audit in 2021 demonstrated that 11 of 47 inpatients (23%) were identified as being on HDAT. Of those, seven instances of HDAT were commenced during review by the multidisciplinary team or the consultant, with only two of these cases noting that the medication prescribed would result in initiating HDAT. Of the remaining cases, the prescriber was unclear. Eight had an ECG within a month prior to commencing HDAT. Only three patients had a repeat ECG within 7 days of initiation. Three patients were noted to gain at least 5 kg in weight following implementation of HDAT.

**Conclusion:**

Education of junior doctors following initial audit had limited impact, likely due to high turnover of doctors. Implementations currently in development include: 1) Departmental teaching session for doctors of all grades, 2) Introduction of stickers on medication charts for patients prescribed HDAT to highlight monitoring recommendations, 3) Development of ward round template to include review of HDAT.

